# Supplementary material for: “How is your thesis going?”–Ph.D. students’ perspectives on mental health and stress in academia
Source: PLoS One. 2023 Jul 3;18(7):e0288103. doi: 10.1371/journal.pone.0288103 (PMC10317224; doi:10.1371/journal.pone.0288103)
Supplement: S1 Table — (DOCX) [file pone.0288103.s001.docx]

**Supporting information S1**

**Table 1. Sample items and descriptives of Ph.D. students (*n* = 589):**

**Percentage (%), mean (*M*), standard deviation (*SD*), minimum and maximum (*Min*-*Max*).**

|  | **%** | ***M* (*SD*)** | ***Min*-*Max*** | ***Median*** | **Missing** |
| --- | --- | --- | --- | --- | --- |
| **Sociodemographics** |  |  |  |  |  |
| Female | 60.3 |  |  |  | 1 |
| Age |  | 28.8 (3.48) | 17-48 | 28 | 5 |
| Children | 8.8 |  |  |  | 1 |
| German | 67.9 |  |  |  | 15 |
| European | 82.9 |  |  |  | 16 |
| **Scientific Discipline**  Science  Humanities  Economic and Social Sciences  Law  Medicine  Theology  Two faculties | 56.7  12.1  10.4  3.2  2.9  2.4  7.0 |  |  |  |  |
| **Ph.D. stage (in months)**  1st year  2nd year  3rd year  4th year | 19.0  22.2  20.4  16.5 | 30.0 (21.3) | 0-133 | 26 | 49 |
| Employment  Permanent employment  Temporary employment  Scholarship  Not employed | 7.6  65.5  12.1  6.5 |  |  |  | 38 |
| Contract length (in months) |  | 34.3 (11.7) | 2-71 | 36 | 197 |
| Percentage of contract |  | 63.0 (15.1) | 10-100 | 65 | 128 |
| **Workload** |  |  |  |  |  |
| Ph.D. work / week |  | 36.0 (15.6) | 0-100^a^ | 40 | 40 |
| Total work / week |  | 44.1 (11.4) | 0-100^a^ | 42 | 47 |

Values do not add up to 100% due to missing/invalid answers.

^a^100 working hours per week are possible. Nevertheless, this might be an outlier and treated carefully.
