# Supplementary material for: “How is your thesis going?”–Ph.D. students’ perspectives on mental health and stress in academia
Source: PLoS One. 2023 Jul 3;18(7):e0288103. doi: 10.1371/journal.pone.0288103 (PMC10317224; doi:10.1371/journal.pone.0288103)
Supplement: S2 Table — (DOCX) [file pone.0288103.s002.docx]

**Supporting information S2**

**Table 2. Used scales and items with percentage (%), mean (*M*), standard deviation (*SD*), minimum and maximum (*Min*-*Max*), *median*, Cronbach’s *alpha*.**

|  | **%** | ***M* (*SD*)** | ***Min*-*Max*** | ***Median*** | **Missing** | ***Alpha*** | **Remark / Source** |
| --- | --- | --- | --- | --- | --- | --- | --- |
| **EV: Evaluation of your Ph.D.** |  |  |  |  |  |  |  |
| EV01 Would Ph.D. start again |  | 3.66 (1.19) | 1-5 | 4 | 90 |  |  |
| EV04 Regret having started Ph.D. |  | 2.22 (1.02) | 1-5 | 2 | 79 |  |  |
| EV06-08 Job Satisfaction Scale |  | 3.35 (0.90) | 1-5 | 3.3 | 55 | 0.86 | (Hellgren et al., 1997) |
| EV09 Life Satisfaction |  | 2.86 (0.98) | 1-5 | 3 | 57 |  |  |
| EV05 Better conditions other university   - yes - no - not sure | 34.7  25.0  40.3 |  |  |  | 50 |  |  |
| **WG: Structure of working group** |  |  |  |  |  |  |  |
| WG01: professional support | 77.8 |  |  |  | 61 |  |  |
| WG02: emotional support | 65.0 |  |  |  | 61 |  |  |
| **GH: General Health** |  |  |  |  |  |  |  |
| GH01-04 Perceived Stress Scale (PSS)  Reported sum score for PSS^a^ |  | 2.95 (0.71)  7.79 (2.86) | 1.00-4.75  0-15 | 3  8 | 67  67 | 0.79 | (Cohen, 1983; Büssing, 2011) |
| **OR: Other responsibilities** |  |  |  |  |  |  |  |
| Supervision of students  Administration  Topic-unrelated research  Others  None | 48.2  56.2  42.4  36.1  7.6 |  |  |  | 66  66  66  66  66 |  |  |
| Time spent for other responsibilities besides research (in %) | 14.1 | 31.2 (22.9) | 1-100 | 25 | 141 |  |  |
| **ST: Stressors** |  |  |  |  |  |  |  |
| ST13-ST15 Job Insecurity Scale |  | 2.92 (1.13) | 1-5 | 3 | 48 | 0.80 | (Hellgren, 1999) |
| Institutional Stressors: Supervision  Positive support: ST02, ST03, ST05, ST07  Negative support: ST01, ST04, ST06, ST08 |  | 3.48 (0.98)  2.18 (0.83) | 1-5  1.00-4.78 | 3.8  2.0 | 77  78 | 0.85  0.76 |  |
| ST17: Mistreated by colleagues |  | 1.80 (0.92) | 1-5 | 2 | 61 |  |  |
| ST09: Regular meetings supervisor |  | 3.41 (1.19) | 1-5 | 4 | 77 |  |  |
| ST16: Frequency meetings |  | 2.38 (1.22) | 1-6 | 2 | 77 |  |  |
| ST11: Worrying long-term contracts |  | 4.25 (1.09) | 1-5 | 5 | 77 |  |  |
| ST12: Find a good job |  | 3.49 (0.97) | 1-5 | 4 | 77 |  |  |
| **MH: Mental Health** |  |  |  |  |  |  |  |
| MH01: How often do you feel stressed since you have started your Ph.D.? |  | 3.55 (0.71) | 1-5 | 4 | 88 |  |  |
| MH02: Has your stress level increased since you started your Ph.D.? | 76.4 |  |  |  | 88 |  |  |
| MH04: Do you have anyone at your institute to consult about your work-related stress?  - Colleagues  - Admins/Coordination  - Supervisor  - Others | 73.7  66.3  5.4  20.5  5.0 |  |  |  | 87 |  |  |
| MH05: Do you feel your mental health has declined due to the Ph.D.? |  | 2.2 (0.97) | 1-4 | 2 | 90 |  |  |
| MH07: Do you know other Ph.D. students who are struggling? |  | 2.6 (0.97) | 1-5 | 2 | 91 |  |  |
| MH08: Do you think MH problems negatively affect your quality of work? |  | 3.3 (1.09) | 1-5 | 3 | 93 |  |  |
| MH09: Would special services help?   - Personalized coaching - Mentoring - Time management - Stress management - Structural changes - Other | 89.8  31.1  44.1  29.9  44.5  62.9  10.6 |  |  |  | 90 |  |  |
| MH10: Psychotherapy | 15.5 |  |  |  | 92 |  |  |
| MH11: Mental disorder | 19.9 |  |  |  | 92 |  |  |
| **PHQ 2: Perceived Health Questionnaire** |  | 2.3 (1.64) | 0-3 | 1 | 82 |  | MH12 & MH14  (Bach et al., 2016) |
| MH12: little interest in doing things |  | 1.2 (0.89) | 0-3 | 1 | 82 |  |  |
| MH14: feeling down, depressed, or hopeless |  | 1.2 (0.91) | 0-3 | 1 | 82 |  |  |
| **GAD 7: General Anxiety Disorder** (from PHQ) |  | 8.5 (4.09) | 1-20 | 8 | 175 |  | Sum of MH15 to MH21  (Bach et al., 2016) |
| MH15: feeling nervous, anxious or on edge |  | 1.1 (0.66) | 0-2 | 1 | 86 |  | If MH15 “not at all”, MH16-MH21 are skipped. |
| MH16: being restless |  | 0.7 (0.86) | 0-3 | 1 | 169 |  | (filter question) |
| MH17: being tired easily |  | 1.7 (0.95) | 0-3 | 1 | 169 |  | (filter question) |
| MH18: muscle pain/tension |  | 1.1 (1.04) | 0-3 | 1 | 171 |  | (filter question) |
| MH19: trouble falling asleep |  | 1.2 (1.02) | 0-3 | 1 | 172 |  | (filter question) |
| MH20: trouble on concentrating |  | 1.2 (0.95) | 0-3 | 1 | 171 |  | (filter question) |
| MH21: becoming easily annoyed |  | 1.2 (0.90) | 0-3 | 1 | 169 |  | (filter question) |
| **SH: Seeking help** |  |  |  |  |  |  |  |
| SH01: tried to improve situation |  | 3.46 (0.94) | 1-5 |  | 96 |  |  |
| SH02: aware of consultation services at university | 54.6 |  |  |  | 91 |  | If SH02 “no”, SH04 is skipped. |
| SH04: how much knowledge about consultation service |  | 2.8 (0.89) | 1-5 |  | 317 |  | (filter question) |
| SH05: Sought for help | 29.2 |  |  |  | 92 |  |  |
| SH06: anyone to talk to | 90.3 |  |  |  | 94 |  |  |
| **COVID-19:** |  |  |  |  |  |  |  |
| SH10: COVID-19 affects general situation   - Improved - Worsened - Affected but not improved/worsened - Not affected | 6.8  41.9  28.5  7.1 |  |  |  | 92 |  |  |
| SH11: COVID-19 affects answers in this survey |  | 2.97 (1.26) | 1-5 |  | 96 |  |  |

Values do not add up to 100% due to missing/invalid answers.

^a^To be able to compare the PSS to other studies, we constructed the sum score which is based on by one the shifted items GH01 to GH04.

For all further analysis we kept the initial range from 1 to 5.
