# Supplementary material for: “How is your thesis going?”–Ph.D. students’ perspectives on mental health and stress in academia
Source: PLoS One. 2023 Jul 3;18(7):e0288103. doi: 10.1371/journal.pone.0288103 (PMC10317224; doi:10.1371/journal.pone.0288103)
Supplement: S3 Table — (DOCX) [file pone.0288103.s003.docx]

**Supporting information S3**

**Table 3. Faculty wise mean comparison on the job insecurity scale.**

| Faculty  (*n*) | 1 .  (334) | 2.  (61) | 3.  (71) | 4.  (17) | 5.  (19) | 6.  (14) | 7.  (41) |
| --- | --- | --- | --- | --- | --- | --- | --- |
| 1. Science | 2.86 (1.13) |  |  |  |  |  |  |
| 2. Economic & Social Sc. |  | 2.88 (1.13) |  |  |  |  |  |
| 3. Humanities | * |  | 3.32 (0.91) |  |  |  |  |
| 4. Medicine |  |  |  | 3.14 (1.22) |  |  |  |
| 5. Law | * | * | ** |  | 2.10 (1.22) |  |  |
| 6. Theology |  |  |  |  |  | 2.38 (1.19) |  |
| 7. Two faculties |  |  |  |  | * | * | 3.24 (1.03) |

*n* = 589. Mean and standard deviation of job insecurity on the diagonal (lower values indicate lower perceived job insecurity, scale 1-5), and significant differences are indicated in the lower triangular part with * *p* < .05. ** *p* < .01 (pairwise comparisons using Wilcoxon rank sum test with continuity correction).
