# Supplementary material for: “How is your thesis going?”–Ph.D. students’ perspectives on mental health and stress in academia
Source: PLoS One. 2023 Jul 3;18(7):e0288103. doi: 10.1371/journal.pone.0288103 (PMC10317224; doi:10.1371/journal.pone.0288103)
Supplement: S4 Table — (DOCX) [file pone.0288103.s004.docx]

**Supporting information S4**

**Table 4. Linear regression model for perceived stress and the predictors.**

| Variable | Estimate | *SE* | *p* |
| --- | --- | --- | --- |
| Perceived Stress (Intercept) | 3.70 | 0.37 |  |
| Age | -0.01 | 0.01 |  |
| Gender | -0.19 | 0.05 | *** |
| Job insecurity | 0.15 | 0.02 | *** |
| Job satisfaction | -0.06 | 0.04 |  |
| Life satisfaction | -0.32 | 0.03 | *** |
| Positive support | 0.02 | 0.04 |  |
| Negative support | 0.13 | 0.04 | ** |

* *p* < .05, ** *p* < .01, *** *p* < .001.
