# Supplementary material for: “How is your thesis going?”–Ph.D. students’ perspectives on mental health and stress in academia
Source: PLoS One. 2023 Jul 3;18(7):e0288103. doi: 10.1371/journal.pone.0288103 (PMC10317224; doi:10.1371/journal.pone.0288103)
Supplement: S5 Table — (DOCX) [file pone.0288103.s005.docx]

**Supporting information S5**

**Table 5. Categories and ratings for the causes of stress.**

| **Item:** [MH03_01] *What is/are the cause(s) of your stress?* | | | | | | |
| --- | --- | --- | --- | --- | --- | --- |
| **Answer Category** | **Kripp.**  **Alpha** | **95% CI** | **Rater**  **1** | **Rater**  **2** | **Mean** | **Deviation** |
| C1: Workload & Time Pressure | 0.76 | 0.70; 0.82 | 213 | 209 | 211 | 53 |
| C2: Supervision (Quality & Quantity) | 0.85 | 0.79; 0.91 | 84 | 93 | 88.5 | 21 |
| C3: Lack of Compensation &  Financial insecurity | 0.82 | 0.70; 0.90 | 33 | 44 | 38.5 | 13 |
| C4: Job Insecurity | 0.86 | 0.80; 0.92 | 88 | 98 | 93 | 21 |
| C5: COVID-19 related regulations | 0.95 | 0.89; 1.00 | 34 | 37 | 35.5 | 3 |
| C6: Pressure to perform | 0.55 | 0.43; 0.66 | 57 | 80 | 68.5 | 52 |
| C7: Family care work | 0.79 | 0.63; 0.92 | 16 | 24 | 20 | 8 |
| C8: Social integration & interactions  (private & professional) | 0.74 | 0.66; 0.81 | 84 | 98 | 91 | 38 |
| C9: Self-perception and sense of success | 0.78 | 0.72; 0.85 | 134 | 131 | 132.5 | 40 |
| C10: Lack of relevant competences & experience (sense) of progress and success | 0.68 | 0.58; 0.78 | 66 | 64 | 54 | 35 |
| C11: Private obligations (excl. family care work) | 0.54 | 0.08; 0.91 | 5 | 6 | 5.5 | 5 |
| C12: Housing market | 0.83 | 0.57; 1.00 | 6 | 6 | 6 | 2 |
| C13: Side-job | 0.71 | 0.50; 0.88 | 13 | 12 | 12.5 | 7 |
| C14: Discrimination | 0.60 | 0.18; 0.90 | 5 | 5 | 5 | 4 |
| C15: Dissertation-related doubts (meaning/ sense of purpose?) | 0.56 | 0.33; 0.77 | 19 | 17 | 18 | 15 |
| C16: Perception of dependence & responsibility | 0.52 | 0.29; 0.72 | 22 | 15 | 18.5 | 17 |
| C17: Lack of external structuring | 0.72 | 0.50; 0.87 | 19 | 14 | 16.5 | 9 |
| C18: Others | 0.26 | 0.16; 0.42 | 43 | 118 | 80.5 | 90 |

The confidence intervals for Krippendorff’s alpha are calculated with a bootstrap sample of 1000.
