# Supplementary material for: “How is your thesis going?”–Ph.D. students’ perspectives on mental health and stress in academia
Source: PLoS One. 2023 Jul 3;18(7):e0288103. doi: 10.1371/journal.pone.0288103 (PMC10317224; doi:10.1371/journal.pone.0288103)
Supplement: S6 Table — (DOCX) [file pone.0288103.s006.docx]

**Supporting information S5**

| **Item: [**MH06_1] *What would need to change to improve your mental health status?* | | | | | | |
| --- | --- | --- | --- | --- | --- | --- |
| **Answer Category** | **Kripp.**  **Alpha** | **95% CI** | **Rater**  **1** | **Rater**  **2** | **Mean** | **Deviation** |
| C1: Compensation & Financial Security | 0.74 | 0.60; 0.86 | 41 | 29 | 35 | 16 |
| C2: Support for Parents | 0.91 | 0.72; 1.00 | 6 | 5 | 5.5 | 1 |
| C3: Psychological Support | 0.91 | 0.81; 0.98 | 24 | 22 | 23 | 4 |
| C4: Manageable Workload | 0.62 | 0.50; 0.74 | 63 | 50 | 56.5 | 35 |
| C5: Supportive Supervision | 0.77 | 0.69; 0.84 | 95 | 102 | 98.5 | 31 |
| C6: Job Security/ Contract | 0.69 | 0.57; 0.78 | 65 | 53 | 59 | 30 |
| C7: Leaving Academia | 0.84 | 0.71; 0.95 | 22 | 18 | 20 | 6 |
| C8: Sense of Achievement/ Experience of Success | 0.44 | 0.06; 0.72 | 14 | 8 | 11 | 12 |
| C9: Less Additional Tasks | 0.78 | 0.64; 0.90 | 30 | 25 | 27.5 | 11 |
| C10: COVID-19 Related Regulations | 0.77 | 0.45; 1.00 | 6 | 7 | 6.5 | 3 |
| C11: Self-perception | 0.70 | 0.51; 0.86 | 17 | 22 | 19.5 | 11 |
| C12: Others | 0.04 | -0.07; 0.10 | 43 | 161 | 102 | 126 |

**Table 6. Categories and ratings for an improvement of mental health.**

The confidence intervals for Krippendorff’s alpha are calculated with a bootstrap sample of 1000.
