# Supplementary material for: “How is your thesis going?”–Ph.D. students’ perspectives on mental health and stress in academia
Source: PLoS One. 2023 Jul 3;18(7):e0288103. doi: 10.1371/journal.pone.0288103 (PMC10317224; doi:10.1371/journal.pone.0288103)
Supplement: S7 Table — (DOCX) [file pone.0288103.s007.docx]

**Supporting information S5**

| **Item:** [SH07_01] *What could be done to improve your situation?* | | | | | | |
| --- | --- | --- | --- | --- | --- | --- |
| **Answer Category** | **Kripp.**  **Alpha** | **95% CI** | **Rater**  **1** | **Rater**  **2** | **Mean** | **Deviation** |
| C1: Pressure to Perform | 0.69 | 0.54; 0.80 | 43 | 36 | 39.5 | 21 |
| C2: Job-Security & Compensation | 0.90 | 0.84; 0.95 | 85 | 86 | 85.5 | 12 |
| C3: Supportive Supervision | 0.87 | 0.79; 0.93 | 69 | 67 | 68 | 13 |
| C4: Manageable Workload | 0.74 | 0.60; 0.86 | 38 | 34 | 36 | 16 |
| C5: Standards & Transparency | 0.61 | 0.27; 0.87 | 7 | 9 | 8 | 6 |
| C6: Services & Support System | 0.70 | 0.56; 0.81 | 38 | 41 | 39.5 | 20 |
| C7: Social Integration | 0.78 | 0.63; 0.90 | 30 | 27 | 28.5 | 11 |
| C8: Support for Parents | 0.82 | 0.59; 1.00 | 9 | 9 | 9 | 3 |
| C9: Power Structure/ PI/ PhD | 0.27 | -0.08; 0.55 | 18 | 14 | 16 | 22 |
| C10. Self-perception | 0.23 | -0.10; 0.54 | 17 | 13 | 15 | 21 |
| C11: Others | 0.40 | 0.22; 0.54 | 39 | 56 | 47.5 | 47 |

**Table 7. Categories and ratings for an improvement of the situation.**

The confidence intervals for Krippendorff’s alpha are calculated with a bootstrap sample of 1000.
